# Hydrogen gas sensing performance of a carbon-doped boron nitride nanoribbon at elevated temperatures

**DOI:** 10.1371/journal.pone.0282370

**Published:** 2023-03-10

**Authors:** Ainun Khairiyah Taib, Zaharah Johari, Shaharin Fadzli Abd. Rahman, Mohd Fairus Mohd Yusoff, Afiq Hamzah

**Affiliations:** School of Electrical Engineering, Faculty of Engineering, Universiti Teknologi Malaysia, Skudai, Johor, Malaysia; Universiti Teknologi Malaysia, MALAYSIA

## Abstract

In this study, computational simulations were used to investigate the performance of a carbon-doped boron nitride nanoribbon (BC_2_NNR) for hydrogen (H_2_) gas sensing at elevated temperatures. The adsorption energy and charge transfer were calculated when H_2_ was simultaneously attached to carbon, boron, and both boron and nitrogen atoms. The sensing ability was further analyzed considering the variations in current–voltage (I–V) characteristics. The simulation results indicated that the energy bandgap of H_2_ on carbon, boron, and both boron and nitrogen exhibited a marginal effect during temperature variations. However, significant differences were observed in terms of adsorption energy at a temperature of 500 *K*, wherein the adsorption energy was increased by 99.62% of that observed at 298 *K*. Additionally, the evaluation of charge transfer indicated that the strongest binding site was achieved at high adsorption energies with high charge transfers. Analysis of the I–V characteristics verified that the currents were considerably affected, particularly when a certain concentration of H_2_ molecules was added at the highest sensitivity of 15.02% with a bias voltage of 3 *V*. The sensitivity at 298 *K* was lower than those observed at 500 and 1000 *K*. The study findings can form the basis for further experimental investigations on BC_2_NNR as a hydrogen sensor.

## 1. Introduction

Gas sensors are extremely essential in today’s society, notably in industrial production and public safety [1]. For detecting various hazardous gases, a high-performance solid-state gas sensor with high sensitivity and selectivity is preferred. Maintaining the stability and selectivity of gas sensors, particularly at higher temperatures, is a challenge in sensing applications [2]. Ideally, high sensitivity, high selectivity, fast response, and rapid recovery time are the desired properties for effective sensing [3, 4].

Metal-based gas sensors were introduced in early 1962; subsequently, Zhang et al. [1] reported different metal oxides involving CeO_2_, ZnO, SnO_2_, and CuO as active materials for hydrogen detection [1]. Over the years, the miniaturization of electronic devices has proposed alternative approaches with respect to device architecture and materials. Graphene, carbon nanotube, silicon nanowire, and other group III-V materials, such as boron nitride (BN) are examples of nanomaterials that commonly used for studying sensors. BN has been increasingly researched owing to its properties similar to those of graphene. Therefore, BN is considered as promising candidate to be utilized in nano-electronic applications [5, 6].

Exposure to gases, such as hydrogen (H_2_), carbon monoxide (CO), hydrogen sulphide (H_2_S), and nitrogen oxide (NO), can pose severe health risks to humans. Moreover, H_2_ is present in other gases such as methane (CH_4_) and ammonia (NH_3_). H_2_ is considered a promising clean energy source and a suitable renewable alternative to fossil fuels [7]. Hydrogen will be a key component of fuel cells, primarily for transportation and automobiles [8]. The acceptance of hydrogen as an alternative energy source reduces pollution, resulting in a change in future climate [9]. Lundström et al. [10] reported the first hydrogen-sensitive metal–oxide–semiconductor field-effect transistor in 1975. Over the years, extensive studies have been conducted on hydrogen sensors owing to their numerous industrial applications. Hydrogen becomes highly flammable and explosive, alternative when mixed with air in a wide range of concentrations exceeding 4% [11–13]. Therefore, hydrogen sensors are extremely essential for leaks detection and combustion control [12, 14].

Numerous studies on hydrogen gas sensing based on experimental works has been done. For examples, Shafiei et al. [15] investigate the electrical characteristic of H_2_ gas sensing with respect to different temperatures and H_2_ gas concentration by using pt/graphene nano-sheets. The concentration of H_2_ gas taken were in the range of 0.06 to 1%, at constant volumetric gas flow rate of 200 *ml*/*min*. The investigation shows at 100°C, a voltage shift of 100 *mV* at 1 *mA* reverse bias current for 1% hydrogen in synthetic air. Another experimental works by Nandi et al. [16] studied the detection on hydrogen gas at room temperature by utilizing plasma-assisted and graphene-functionalized core-shell assembly of SnO_2_ Nanoburflower. Whilst Zhang et al. [1] studied hydrogen gas sensor based on metal oxide nanoparticles decorated with SnO_2_ NPs on graphene transistor. The studied reveals when hydrogen concentration is 100 *ppm*, it exhibits fast response, high sensitivity and short recovery times about 1 *s* at 50°C. Meanwhile Al-Hardan et al. [17] reported a study on sensing mechanism of hydrogen gas sensor in the range of 200−1000 *ppm* based on RF-sputtered ZnO thin films. The findings showed, I-V characteristics revealed an ohmic behaviour of the contacts to the sensor and the complex impedance spectrum (IS) showed a single semicircle with shrinkage in the diameter when the temperature is increased. Matsuura et al. [18] successfully found that Pt-WO_3_/Si microring resonator able to detect hydrogen gas as low at concentration of 0.1 *vol*% with a response time of 10 *s* at each temperature of 5, 20, *and* 30°C. The experiment also revealed gas sensitivity increased when temperature is decreasing. Further study by Ibrahim et al. [19] demonstrated Pt/g-C_3_N_4_ able to show a good sensitivity in sensing hydrogen gas in terms of fast response and recovery time. Other findings on experimental works provide by Lee et al. [7] investigate the performance for hydrogen gas on sputtered PdO decorated TiO_2_ sensing layer. The hydrogen gas concentration of 100, 200, *and* 400 *ppm* was applied to different PdO thickness of 3, 4.5, 6.5 *and* 13 *nm* on the TiO_2_ film. The sensitivity as 6 μ*V*/*ppm* for 3 *nm* PdO decorated TiO_2_ sensor was found under 33 *mW* power consumption. Further, Steinebach et al. [20] investigated the performance of hydrogen gas sensing for NiO ranging from 500 to 10,000 *ppm* at high operating temperatures in gas mixtures such as H_2_, NO_x_, NH_3_ and CO_2_. They observed more than 50% reduction in the gas sensor response with exposure to pure H_2_ and the selectivity shows decreasing with increasing in temperature in a mixture of H_2_ and NO_x_ or NH_3_.

As much as previous studied has done based on experimental works, there is also studies carried in simulation works. For examples, a first-principles study on hydrogen gas sensing of carbon-doped ZnO nanotube by Rezaie et al. [21]. It was found, hydrogen gas is chemically absorbed to the carbon atoms and shows high response with sensitivity of 73% at *V* = 3.5 *V* on the outside and inside of carbon-doped ZnO nanotube. Another simulation works focusing on hydrogen gas binding energies on carbon-doped BN nanotube demonstrated by Baierle et al. [22]. Subsequently, the binding energies reported to have similar value of −156 *meV* and −163 *meV* for H_2_ molecule absorbed on BNNT + C_B_ and BNNT + C_N_, respectively. Moreover, a donor and acceptor defect level were shown when H_2_ molecule absorbed on BNNT + C_B_ and BNNT + C_N_, respectively. Apart from that, Yu et al. [23] studied the potential of monolayer Ti_2_CO_2_ as gas sensor in detecting NH_3_, H_2_, CH_4_, CO, CO_2_, N_2_, NO_2_ and O_2_ gas. Among the gases tested, NH_3_ was found chemisorbed and have the high sensitivity and selectivity with highest adsorption energy of −0.37 *eV*. By applying 3% strains on Ti_2_CO_2_, NH_3_ have the high sensitivity and selectivity with highest adsorption energy of −0.51 *eV*. Another successfully simulation work performed by Zhou et al. [24], studied the monolayer WS_2_ upon adsorption and the impacts of charge transfer mechanism on Fermi-level pinning in sensing H_2_, O_2_, H_2_O, NH_3_, NO, NO_2_ and CO gases. Through this, O_2_, H_2_O, NH_3_ and NO_2_ gases were seen to be a promising candidate as a sensor to WS_2_ with moderate adsorption energies and charge transfer.

BN is known for certain properties, such as a wide band gap of approximately 5−6 *eV*, excellent mechanical strength, high thermo-conductivity, high thermal stability, appropriate chemical inertness, adequate oxidation stability (up to 800°C), and suitable optical properties [25]. Numerous studies have used BN for sensing applications. According to Sajjad et al. [6] that carbon-based band gap tailoring approaches significantly enhance the band gap. The alloying process involving C, B, and N atoms has led to the formation of certain configurations, such as hexagonal boron carbonitride (BxCyNz) and BCN. Based on our literature review, we found number of works has been done investigating nanomaterial for use in hydrogen gas sensing application as in reference [15, 22]. The emerging of nanomaterial such as graphene, carbon nanotube, phosphorene has open possibility of designing a sensitive gas sensor. Previous work as in reference [22] shows that this kind of nanomaterial have higher binding energy. Baierle et al. [22] indicate the introduction of carbon substitution increases the binding energy substantially. The material structure of graphene, phosphorene and carbon nanotube are almost the same with some common characteristic due to the hexagonal bond between the molecules. Likewise, BC_2_N, it has the same hexagonal structure. Considering that BCxN has been increasingly investigated [26, 27], the BC_2_N compound was selected for the analysis in this study. Moreover, as BC_2_N has not been explored as a hydrogen gas sensor thus far, its analysis for H_2_ gas sensing applications is essential. We are motivated to investigate the possibility of applying BC_2_N for use in sensing hydrogen gas. With this we can benchmarked the performance with other nanomaterials.

To determine the feasibility of carbon-doped boron nitride nanoribbon (BC_2_NNR) for sensing H_2_ gases during temperature variations, including at room temperature, the performance reaction of H_2_ was analyzed in this study considering different temperature settings. Herein, BC_2_NNR with armchair edges was used for sensing, and its electronic properties were investigated based on the band structure. The sensing ability was assessed by analyzing the adsorption energy, charge transfer, and sensitivity. The simulation results can form the basis for further experimental analyses on hydrogen gas sensors.

The remainder of this paper is organized as follows. The computational details of the simulation are described in Section 2. Section 3 presents the obtained results and discusses their inferences. Finally, the conclusions of the study are summarized in Section 4.

## 2. Methodology

**Fig 1:**
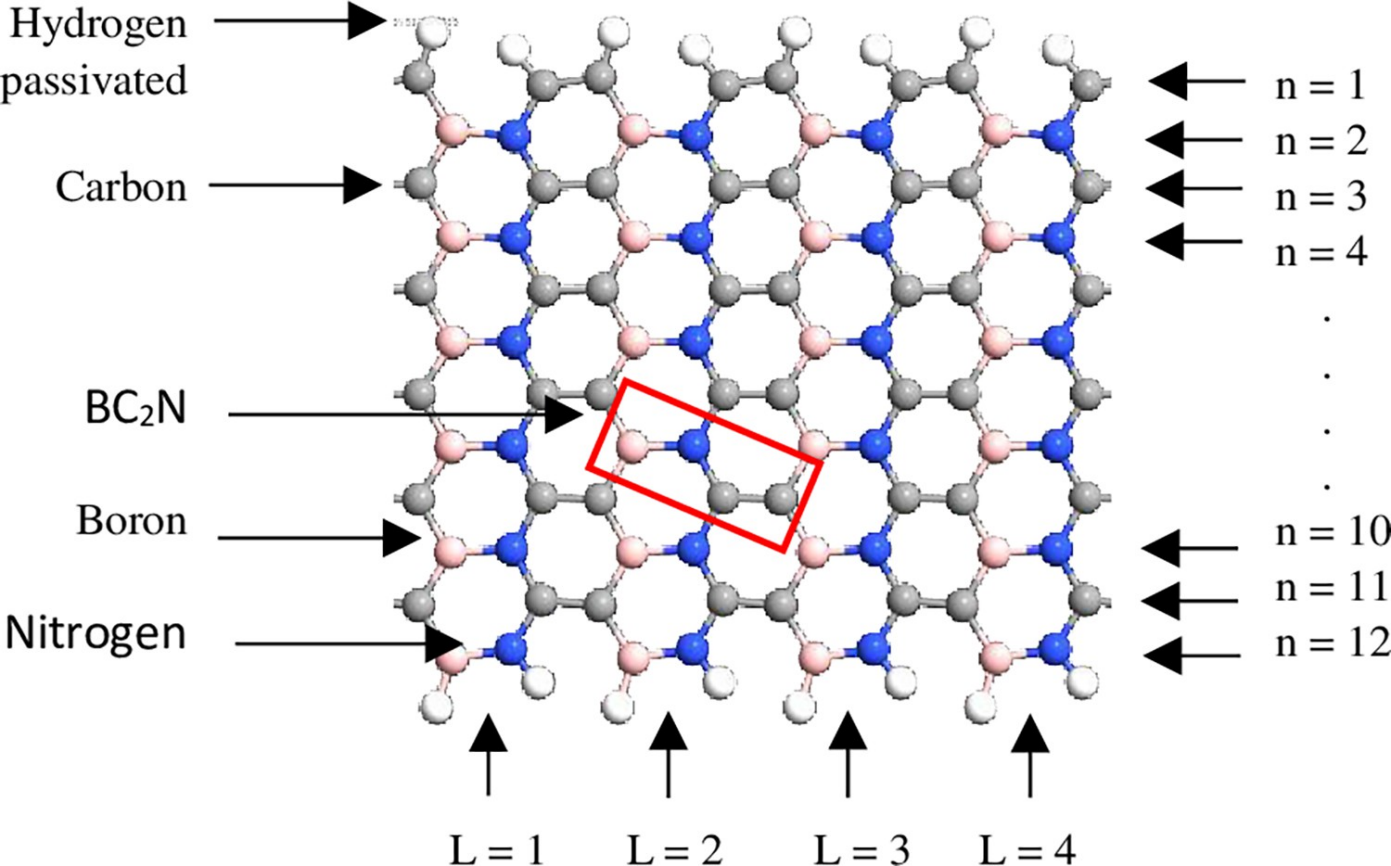
Optimized structure of the pristine A-BC_2_NNR with n = 12.

**Fig 2:**
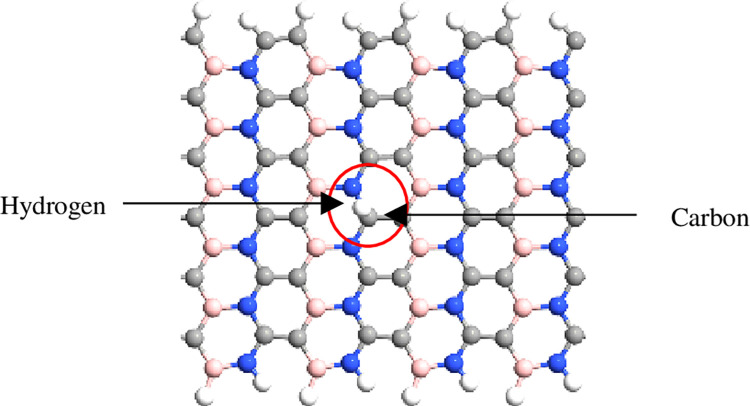
Optimized structures of 12-BC_2_NNR with different positions of H_2_ gas molecules at carbon atom.

**Fig 3:**
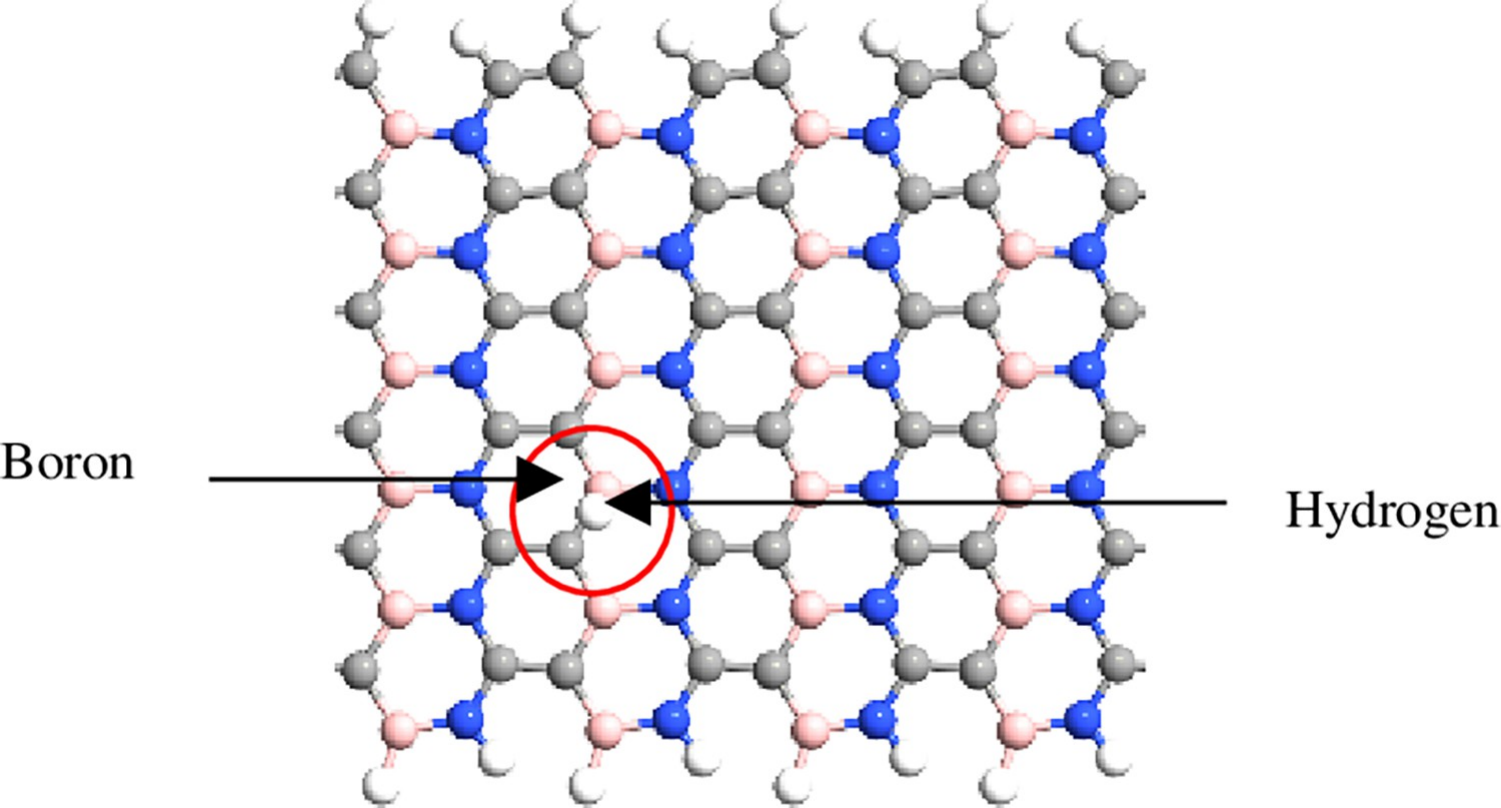
Optimized structures of 12-BC_2_NNR with different positions of H_2_ gas molecules at boron atom.

**Fig 4:**
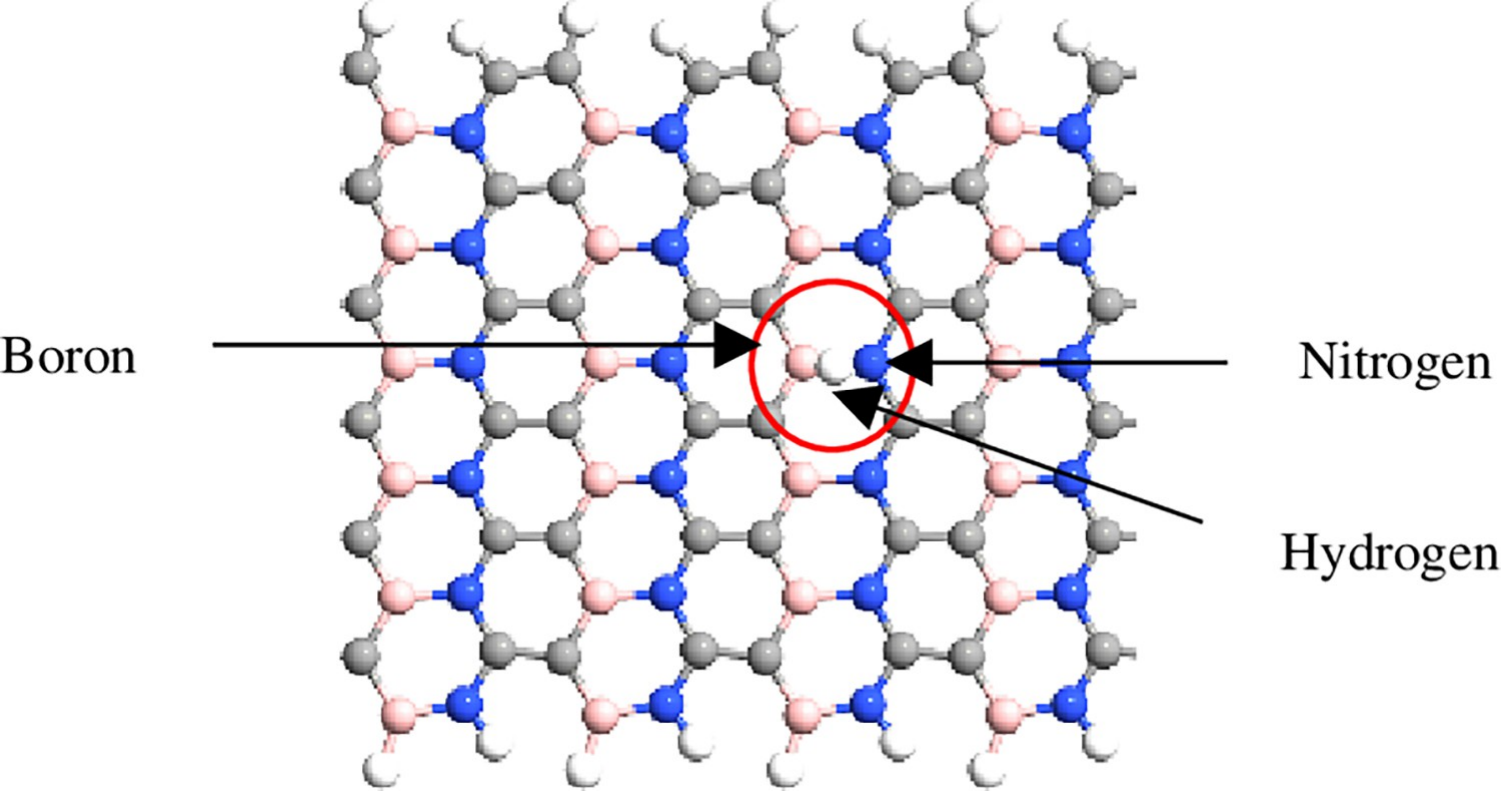
Optimized structures of 12-BC_2_NNR with different positions of H_2_ gas molecules at both boron and nitrogen atoms.

**Fig 5:**
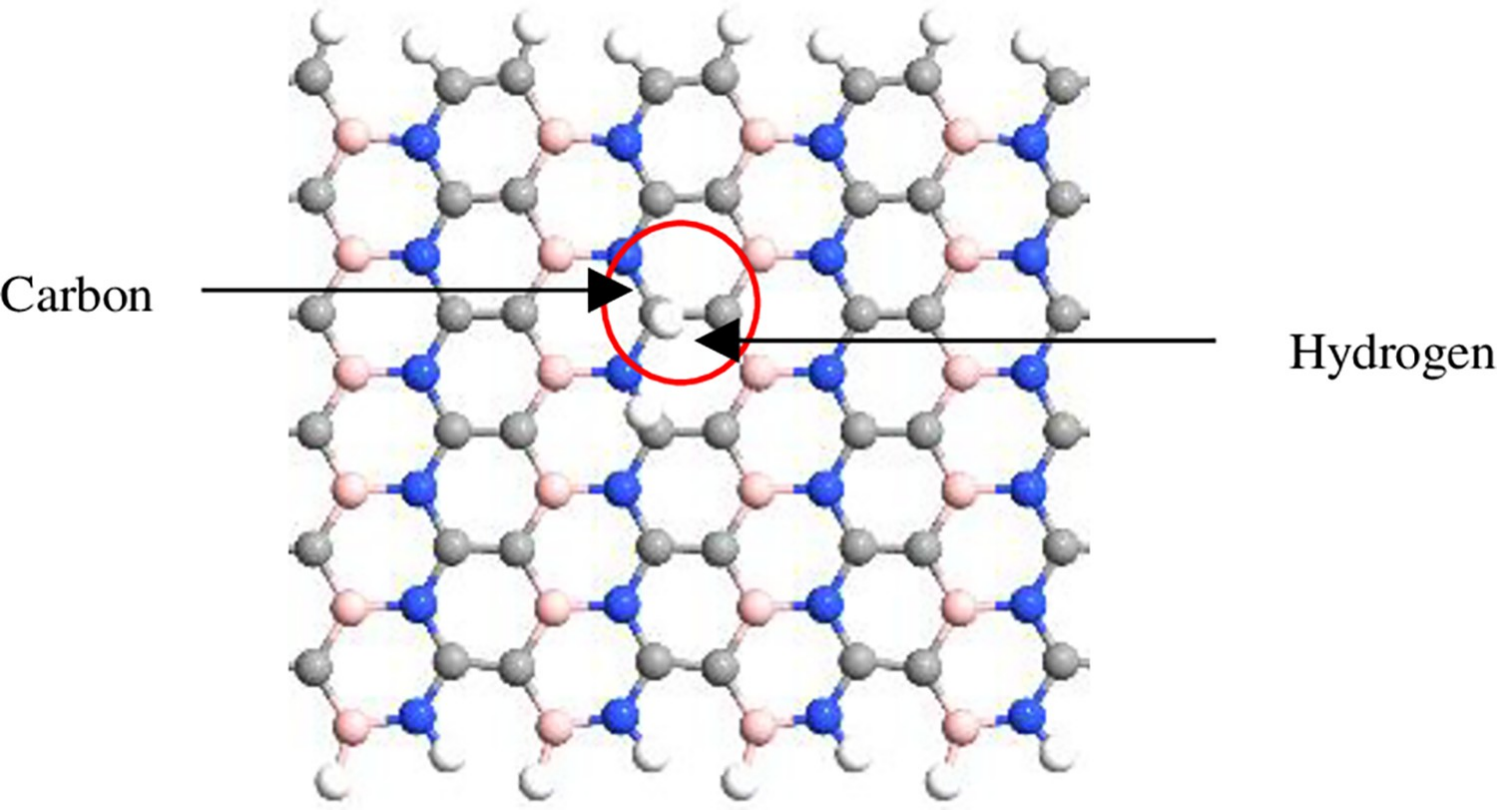
Optimized structures of 12-BC_2_NNR with multiple H_2_ gas molecules in different positions at carbon atom.

**Fig 6:**
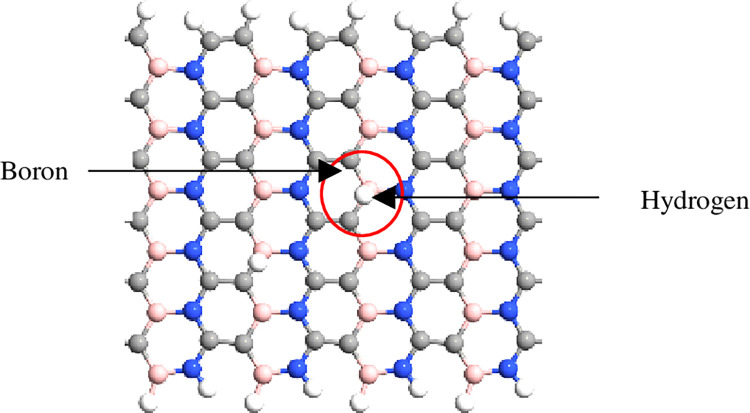
Optimized structures of 12-BC_2_NNR with multiple H_2_ gas molecules in different positions at boron atom.

**Fig 7:**
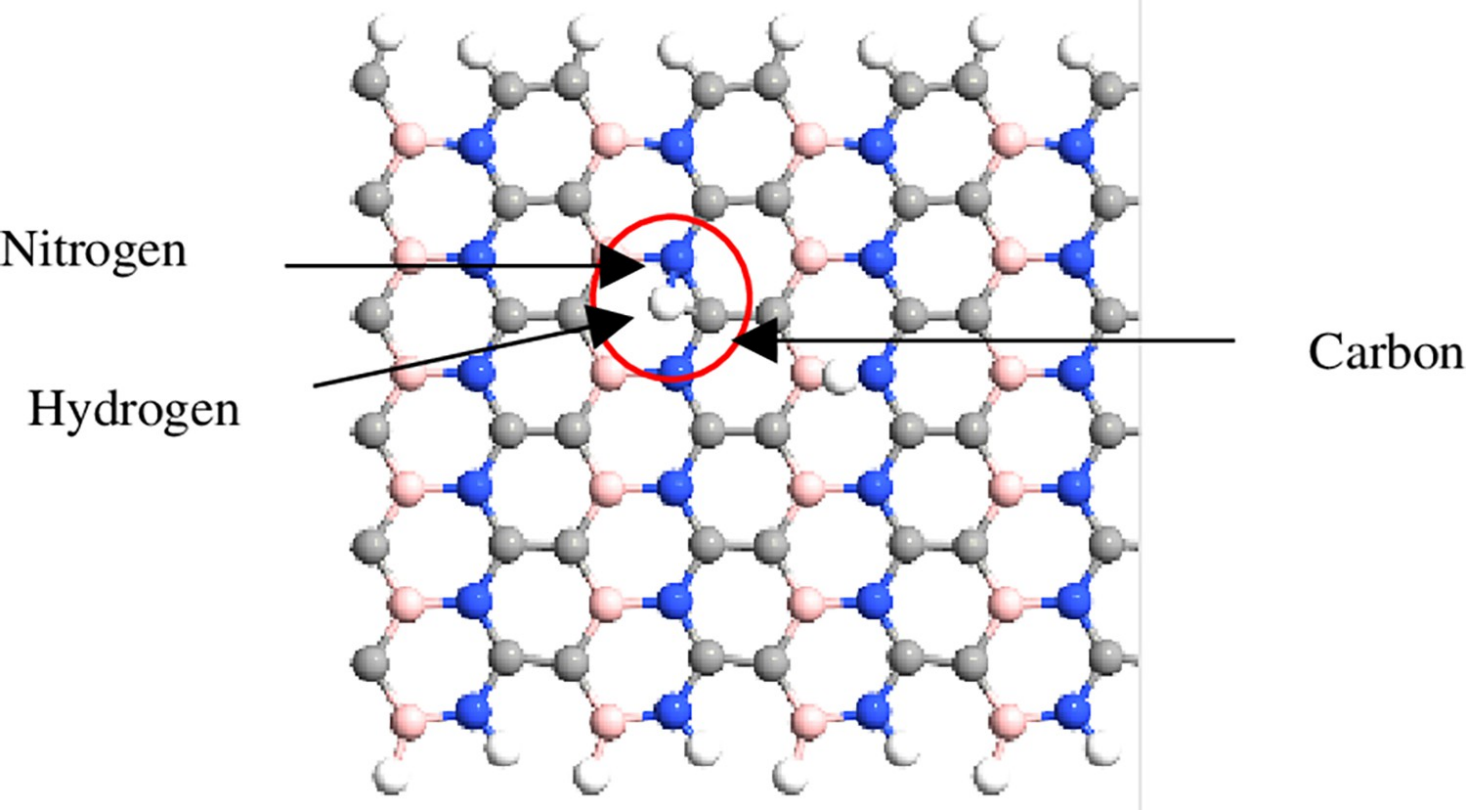
Optimized structures of 12-BC_2_NNR with multiple H_2_ gas molecules in different positions at carbon and nitrogen atoms.

The simulation was performed using QuantumATK from Synopsys (version S-2021-06). Carbon atoms were added within the structure of BN to obtain the BC_2_N structure (Fig 1) with different numbers of dimer lines (n), namely, 4, 6, 8, 10, *and* 12. Subsequently, four-unit cells representing length, L were repeated along the z-axis and maintained constant. The arrangement of B-N-C-C along the armchair lines in this study was based on a previously reported study [28]. Figs 2–4 depicts three additional models with different positions of H_2_ molecules attached to 12-BC_2_NNR. In general, the H_2_ gas molecules were attached to the top of BC_2_N over carbon (Fig 2), boron (Fig 3), and both boron and nitrogen atoms (Fig 4). Figs 5–7 depicts the structures obtained by multiplying the H_2_ molecules in different positions. As indicated in the figure, another H_2_ molecule was added at a different location and attached to the top of BC_2_N over carbon (Fig 5), boron (Fig 6), and both carbon and nitrogen atoms (Fig 7). Generally, the structures of H_2_ molecules illustrated in Figs 2–4 were considered for the analysis. All structures exhibited an initial bond length of 1.48 Å for A-BC_2_NNR. The H_2_ molecules for the three models with different positions were randomly attached at varying distances to analyze the gas adsorption. For H_2_ gas of the 12-BC_2_NNR structure located directly on the carbon, boron, and both boron and nitrogen atoms, the bond lengths were 1.13, 1.21, 1.92, and 1.96 Å, respectively. Furthermore, the distances for the addition of H_2_ molecules were 1.16, 1.4, 1.16 and 1.17 Å, for carbon, boron and both carbon and nitrogen atoms, respectively. Finally, the distance of the H-H gas molecule was maintained at 0.64 Å.

All calculations were performed using the linear combination of atomic orbitals-based density functional theory. The exchange-correlation electron was computed using the Perdew–Zunger formalism of local density approximation. Furthermore, the double-zeta polarized basis set was used for all calculations. To ensure numerical accuracy, the density mesh cut-off energy was set to 75 Hartree. The Brillouin zone (BZ) was sampled using a k-point sampling of 1×1×100 for the electronic structure calculation. Initially, the geometries of all atomic structures in the unit cells were optimized using the limited-memory Broyden–Fletcher–Goldfarb–Shanno algorithm optimization method. LBFGS is the recommended optimizer to use in QuantumATK. In addition, it is necessary to undergone geometry optimization as it will relax the structure and find the minimum energy structure in the modeled structure in order to obtain accurate results. A vacuum region of 10 Å was added along the non-periodic boundary to prevent artificial inter-ribbon interactions. Thus, the sensing performance of H_2_ on BC_2_N was investigated during temperature changes to maximize its functionality. The temperatures selected for the evaluation were 298, 500, and 1000 *K*.

## 3. Results and discussion

**Fig 8:**
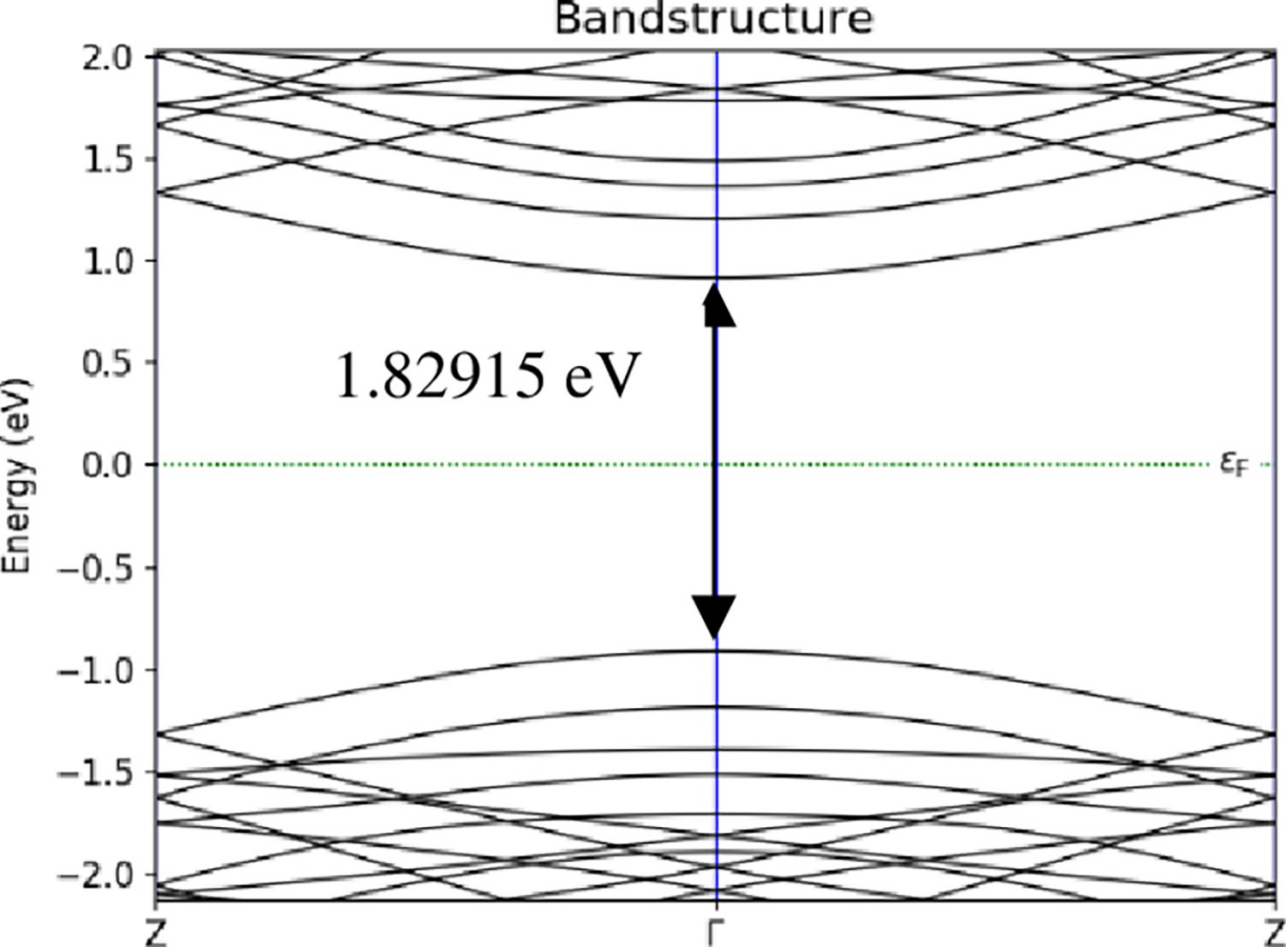
Energy band structures of 12-BC_2_NNR with different positions of H_2_ gas molecules at carbon atom for *T* = 298 *K*.

**Fig 9:**
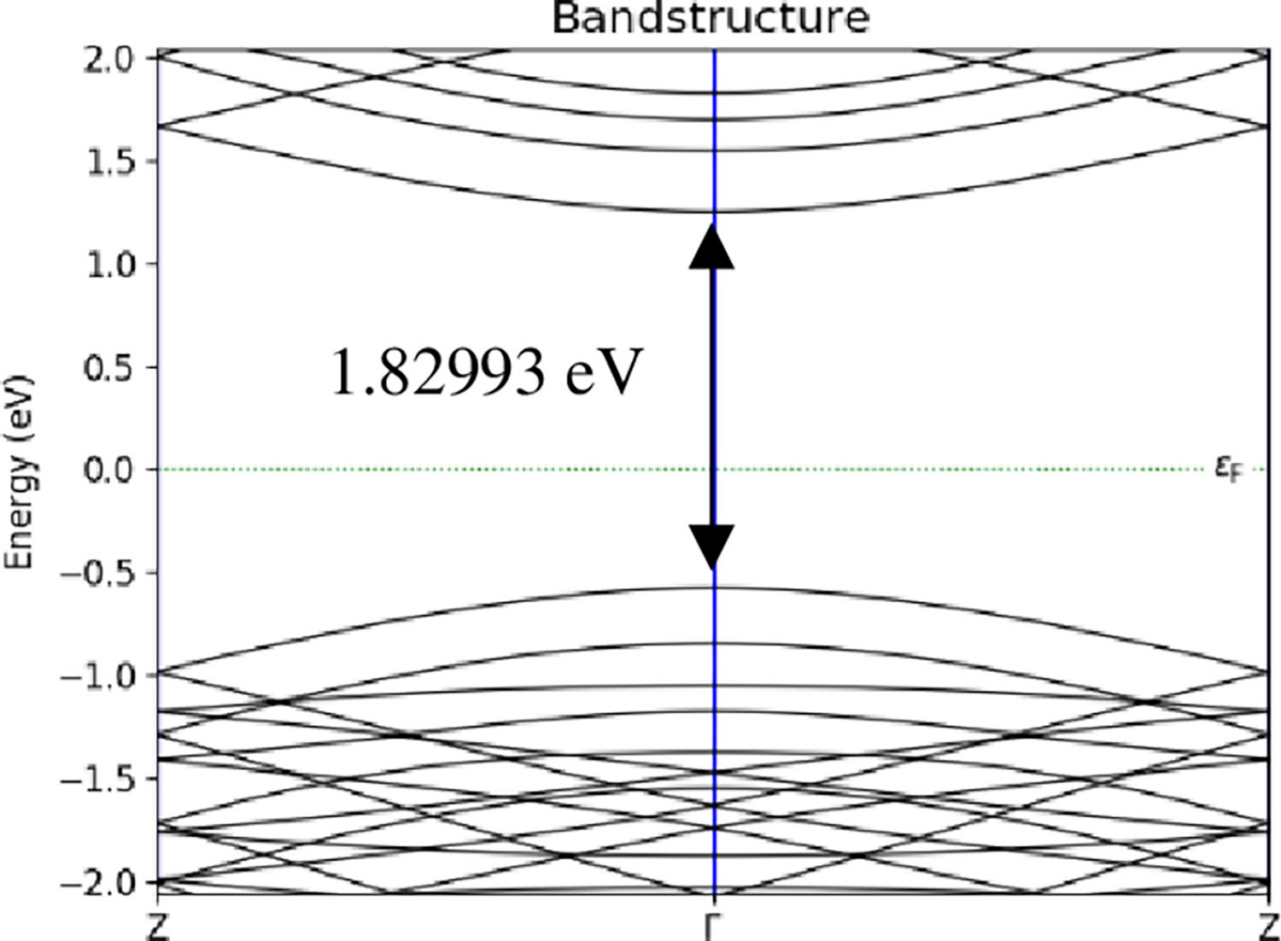
Energy band structures of 12-BC_2_NNR with different positions of H_2_ gas molecules at boron atom for *T* = 298 *K*.

**Fig 10:**
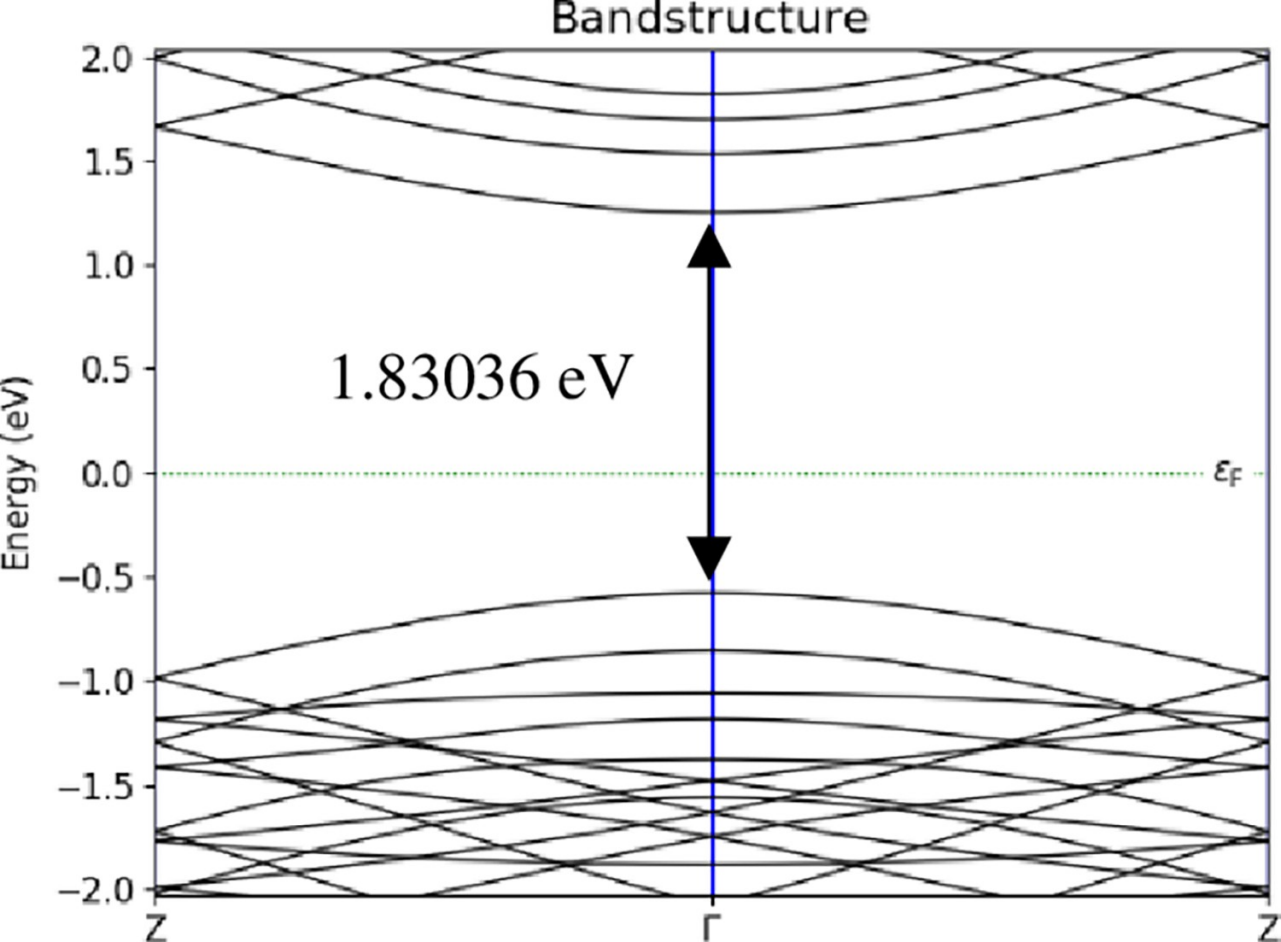
Energy band structures of 12-BC_2_NNR with different positions of H_2_ gas molecules at both boron and nitrogen atoms for *T* = 298 *K*.

The electronic properties were focused on the energy band structure, adsorption energy, and charge transfer. Meanwhile, the transport properties were focused on I-V characteristic to calculate the sensitivity. In the simulation of the energy band structure, A-BC_2_NNR followed the BZ route from Z point to Γ and back to Z point. Figs 8–10 depicted sample of the energy band structure for *T* = 298 *K*. Table 1 lists the evolution of the energy band gap for the three temperatures considered in the study at three different positions of H_2_ gas molecules attached to the surface of 12-BC_2_NNR; the simulation was performed to determine whether the energy band gap would alter.

**Table 1:** Comparison of energy band gap on the 12-BC_2_NNR surface at three different temperatures.

Position	T = 298 K	T = 500 K	T = 1000 K
Carbon	1.82915 *eV*	1.83193 *eV*	1.83017 *eV*
Boron	1.82993 *eV*	1.83095 *eV*	1.82946 *eV*
Boron and nitrogen	1.83036 *eV*	1.83302 *eV*	1.83432 *eV*

The energy band gap was observed in the gap between the conduction and valence bands using the band structure process. According to Table 1, the energy band gap openings at all temperatures for the three different positions of H_2_ gas molecules were similar (approximately 1.83 *eV*). The changes in the energy band gap were similar, indicating that the shift in the lower conduction band and the upper valence band were independent of the temperature changes. However, it can be noticed in Figs 8–10 above, each position of H_2_ gas have difference number sub-band lines in both conduction and valence bands. Furthermore, the attachment position of the gas molecule was not a sensitive factor affecting the energy band gap of BC_2_N.

**Fig 11:**
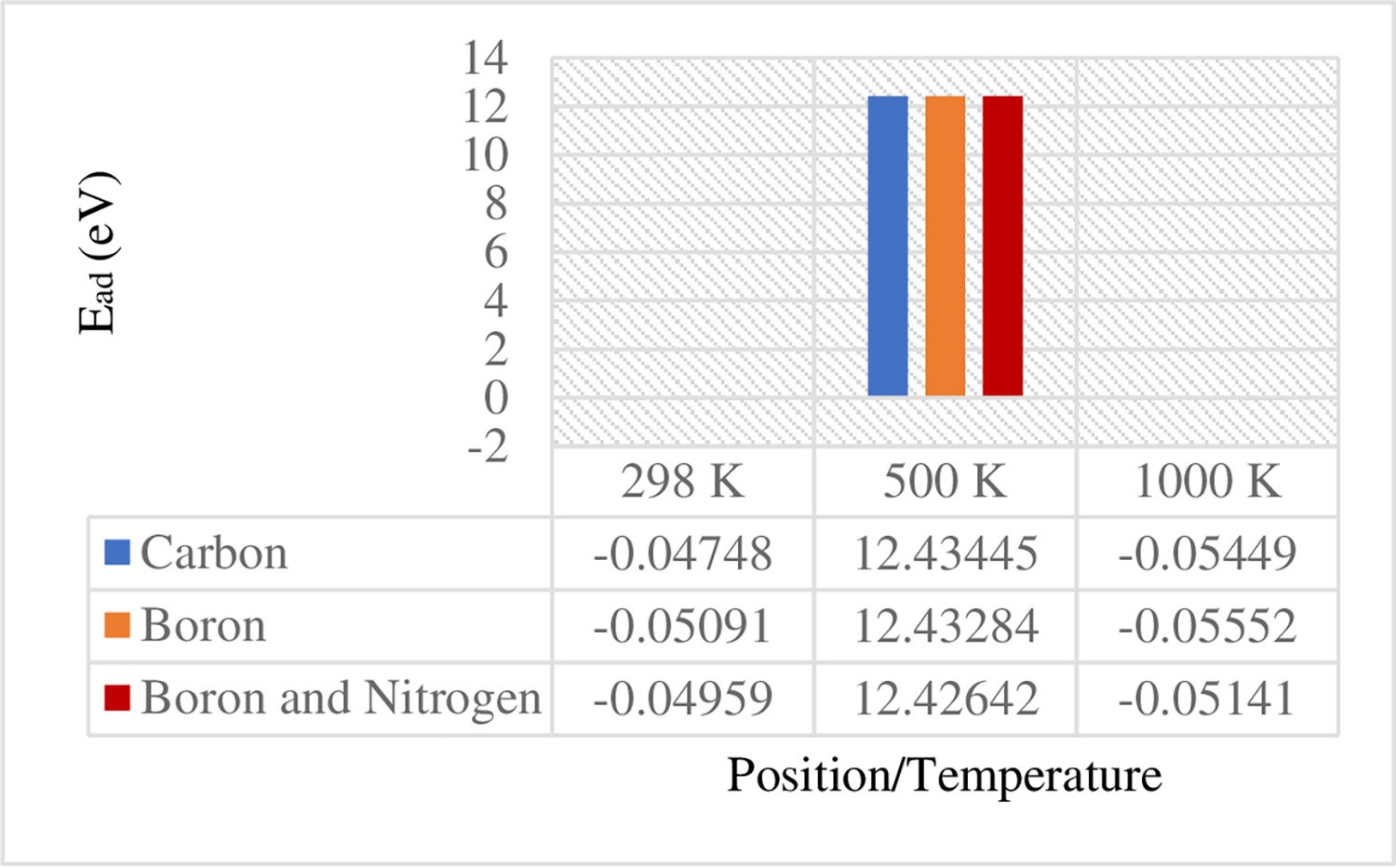
Comparison of the adsorption energy for 12-BC_2_NNR at three different temperatures.

**Fig 12:**
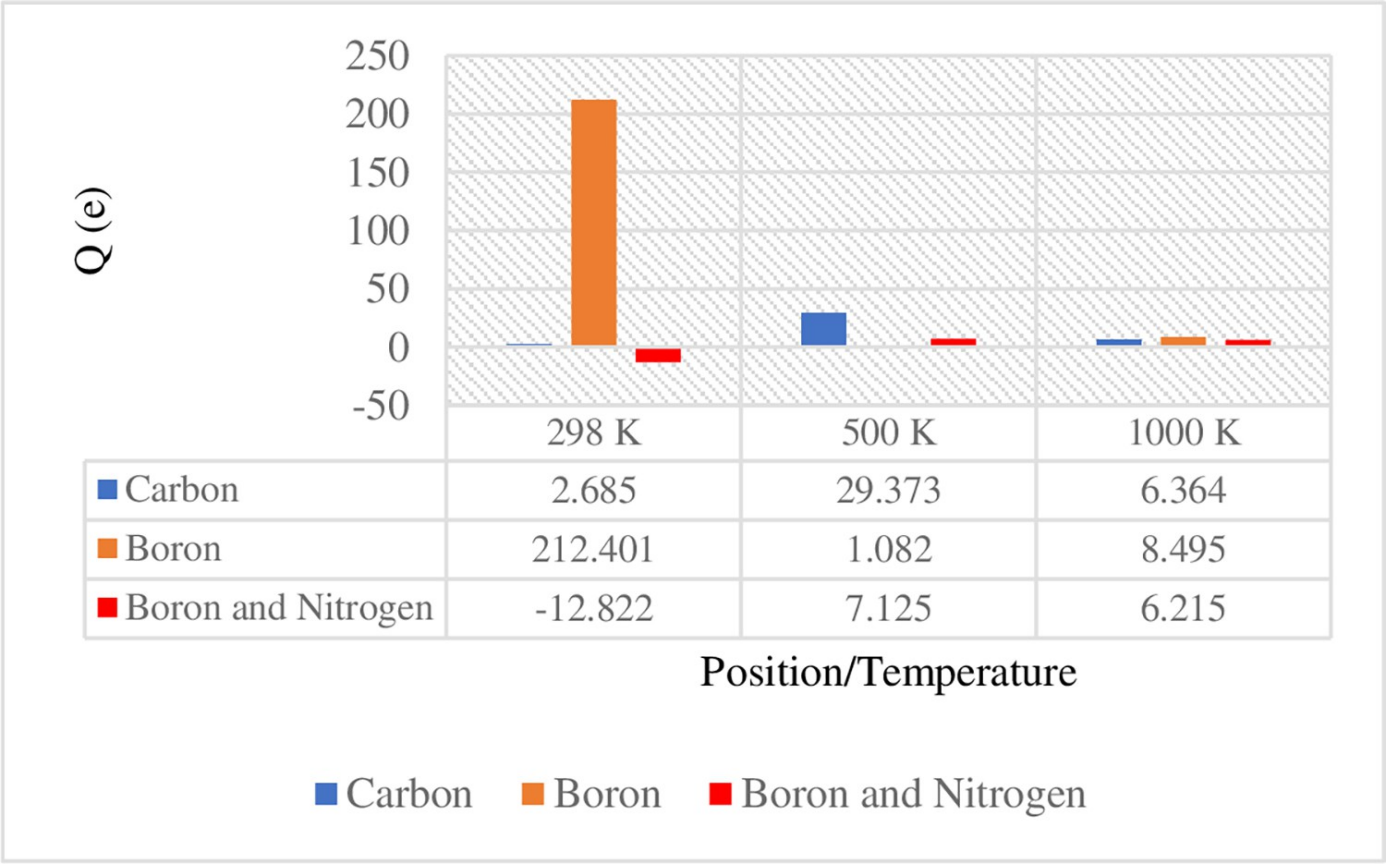
Comparison of the charge transfer for 12-BC_2_NNR at three different temperatures.

To further understand the potential of BC_2_NNR as a gas sensor, the adsorption energy, charge transfer, and sensitivity were simulated. The adsorption energy (E_ad_) was calculated using Eq (1) to measure the strength of H_2_ gas sensing bonds with BC_2_N [29, 30].

$$E_{ad}=E({BC}_{2}N+H_{2})-E({BC}_{2}N)-E(H_{2}),$$ (1)

where *E*(*BC*_2_*N*+*H*_2_) denotes the total energy of BC_2_NNR with an absorbed H_2_ gas molecule, *E*(*BC*_2_*N*) indicates the total energy of BC_2_NNR, and *E*(*H*_2_) represents the total energy of the isolated H_2_ gas molecules. The charge transfer (Q) was evaluated using the basic set method, as indicated in Eq (2), using the Mulliken population (N) [31–33].

$$Q=Q_{a}-Q_{b},$$ (2)

where *Q*_*a*_ *and Q*_*b*_ denote the net Mulliken charges of a gas molecule after and before its adsorption, respectively. Figs 11 and 12 depict the comparison of the adsorption energy and charge transfer data, respectively, in terms of the attachment position of gas molecules at elevated temperatures, varying from 298 to 1000 *K*.

To analyze the gas sensing mechanism at room temperature (25°C), 298 *K* was considered as one of the temperature values for the evaluation. Among the three positions, the strongest binding site was identified in the case of the hydrogen atom located directly above the boron atom (Fig 3), with an adsorption energy of −0.05091 *eV*. A significant transfer of electrons of 212.401 *e* was observed from H_2_ gas molecules to BC_2_N. This is noticeable different on the charge transfer when hydrogen molecules on boron atom. According to the energy band structure in Fig 9 above, more localized states produced below Fermi level in position of boron atom compared to carbon and both boron and nitrogen atoms. This is indicated that large amount of charge acceptor of 212.401 *e* was accumulated at valence band state. Thus, this signify BC_2_N received a large number of electrons from the H_2_ gas. As mentioned by Zhou et al. [24], charge transfer contributor should be larger in order for it to sensing. Minimum increments of 6.7 and 2.6% were observed in the adsorption energy and charge transfer, respectively, from the positions of hydrogen on carbon and both boron and nitrogen atoms. Table 2 below listed the comparison data recorded from previous studies on the adsorption energies and charge transfer from H_2_ gas molecules to different materials.

**Table 2:** The adsorption energies and charge transfer from H_2_ gas molecule to different materials.

Material	E_ads_	Q
Ti_2_CO_2_ [23]	−0.005 *eV*	0.003 *e*
WS_2_ (H site) [24]	−0.068 *eV*	−
WS_2_ (Tw site) [24]	−0.075 *eV*	0.002 *e*
WS_2_ (Ts site) [24]	−0.037 *eV*	−
WS_2_ (B site) [24]	−	−
BNNT + C_B_-H_2_ [22]	−0.156 *eV*	−
BNNT + C_N_-H_2_ [22]	−0.163 *eV*	−

Similar observations were reported by Yu et al. [23], where the adsorption energy from H_2_ gas molecules to Ti_2_CO_2_ was achieved at −0.05 *eV* and a charge transfer of 0.003 *e* was observed [23]. The negative sign of the adsorption energy indicated stronger adsorption and stability [32] with the exothermic reaction [34]. For the case of WS_2_ the top of a W atom (Tw), has the most favorable site with −0.075 *eV* with smallest charge transfer contributions with 0.002 *e* [24]. The substitution impurities of carbon atom in different sites of BNNT has shown physisorption occurs with no preference for H_2_ molecule to absorbed as very close adsorption energies was recorded of −0.156 *and* −0.163 *eV* [22]. Apparently, result showing in carbon-doped BNNT is larger than in presented work, BC_2_NNR. This could be due to the different in dimensional models of BN nanomaterial and to its chemical properties’ reaction. Salih et al. [32] reported that a negatively charged value of charge transfer indicates the electron loss of the gas. In this study, a negative charge was observed only at a temperature of 298 *K* at both boron and nitrogen atoms (Fig 12).

At 500 *K*, the preferable binding site was observed between the hydrogen molecule and carbon atom, with an adsorption energy of 12.43445 *eV* and a charge transfer of 29.373 *e* from H_2_ gas molecules to BC_2_N. Here, only infinitesimal increments of 0.01 and 0.06% were observed in the adsorption energy and charge transfer, respectively, from boron and both boron and nitrogen atoms. As indicated in Fig 11, the adsorption energies of two positions on BC_2_N were slightly greater than 12.43445 *eV*, ranging from 12.42642 to 12.43284 *eV*. This was also observed in the case of charge transfers between the H_2_ gas molecules and BC_2_N, which were smaller than 29.373 *e*, ranging from 1.082 to 7.125 *e*. According to Salih et al. [32], the researcher found that the negative sign of the adsorption energy indicated stronger adsorption and stability. Thus, referring to the significant adsorption energy recorded, the adsorption energy observed for temperature of 500 *K* were seen as not stronger and stable, although the preferable binding was recorded. This can be realized that, the positive sign achieved for all three positions.

By contrast, the strongest binding site at 1000 *K* was identified at the position of boron with an adsorption energy of −0.05552 *eV* and a charge transfer of 8.495 *e* from H_2_ gas molecules to BC_2_N. The adsorption energies of two positions (at carbon and both boron and nitrogen) on BC_2_N ranged from −0.05141 to −0.05449 *eV*, whereas the charge transfer ranged from 6.215 to 6.364 *e*, which were smaller than that obtained at the position of boron. The adsorption energy at the position of boron was increased by 7.4 and 1.9% compared to those at the positions of the boron and nitrogen and carbon atoms, respectively. The comparison of the elevated temperature changes from 298 to 1000 *K* (Fig 11) revealed that the adsorption energy was the highest for all three positions at 1000 *K*.

According to Guo et al. [35], the adsorption energy should be greater than −0.50 *eV* to effectively capture gas molecules on solid surfaces. As indicated in Fig 11, none of the adsorption energy observed in this study was greater than −0.50 *eV*. Additionally, Guo et al. [35] reported that the perfect h-BN sheet is unsuitable for gas separation and adsorption at room temperature. This could be the reason for the adsorption energy not effectively capturing the H_2_ gas molecule on BC_2_N at 298 *K*. In addition, BC_2_N was seen as unsuitable for sensing H_2_ gas due to its relatively small adsorption energy, approximately taken at −0.05 *eV* and low binding energy was reported between BC_2_N and H_2_ gas.

To further evaluate the potential of BC_2_N as a hydrogen gas sensor, the sensitivity was calculated using currents obtained from the current–voltage (I–V) characteristics with and without exposure to gas molecules, as indicated in Eq (3) [36].

$$Sensitivity\%=\left|\frac{I-I_{O}}{I_{O}}\right|\times 100,$$ (3)

where *I* and *I*_*O*_ denote currents from the system with and without the H_2_ gas molecules, respectively; both currents were calculated at the highest bias voltage of 3 *V*. Table 3 lists the comparison of sensitivity based on the attachment positions at temperatures varying from 298 to 1000 *K*.

**Table 3:** Comparison of sensitivity for 12-BC_2_NNR at three different temperatures.

Position	Sensitivity %		
	T *=* 298 K	T *=* 500 K	T *=* 1000 K
Carbon	1.83	2	3.38
Boron	18.54	18.76	71.36
Boron and nitrogen	2.17	1.63	1.3

**Fig 13:**
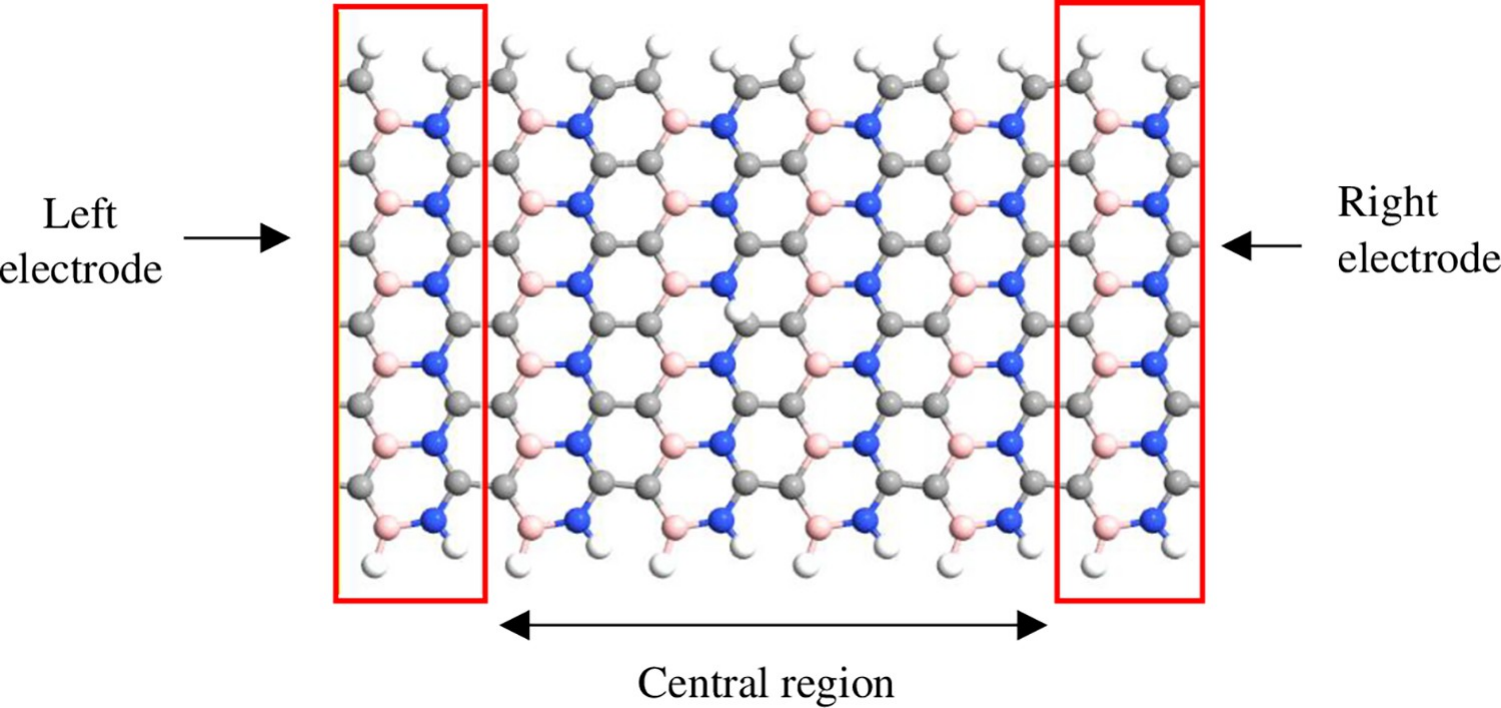
BC_2_N-based sensor connected with two electrodes for detecting H_2_ gas molecules at the position of carbon.

Fig 13 depicts the two electrodes which are left and right electrodes that connected to BC_2_N to evaluate its performance as a hydrogen sensor. The I–V characteristics before and after the adsorption of H_2_ gas molecules were simulated considering the three temperatures. The construction of the two electrodes are implemented in QuantumATK software under plugin features tool called device from bulk. Initially, the nanoscale structure needed for QuantumATK for transport calculations consists of three main parts; left electrode, central region and right electrode as depicted in Fig 13. In a transistor perspective, the left and right bulk (periodic) electrodes are connected to the device’s central region, which is finite (non-periodic) along the transport direction. The central region is also known as the scattering region because changes in the physical and chemical environment in the central region scatter charge carriers travelling between two electrodes. Furthermore, the central region determines the device’s functionality, whereas the left and right electrodes are modelled as perfect leads connecting the device to the external source and drain.

**Fig 14:**
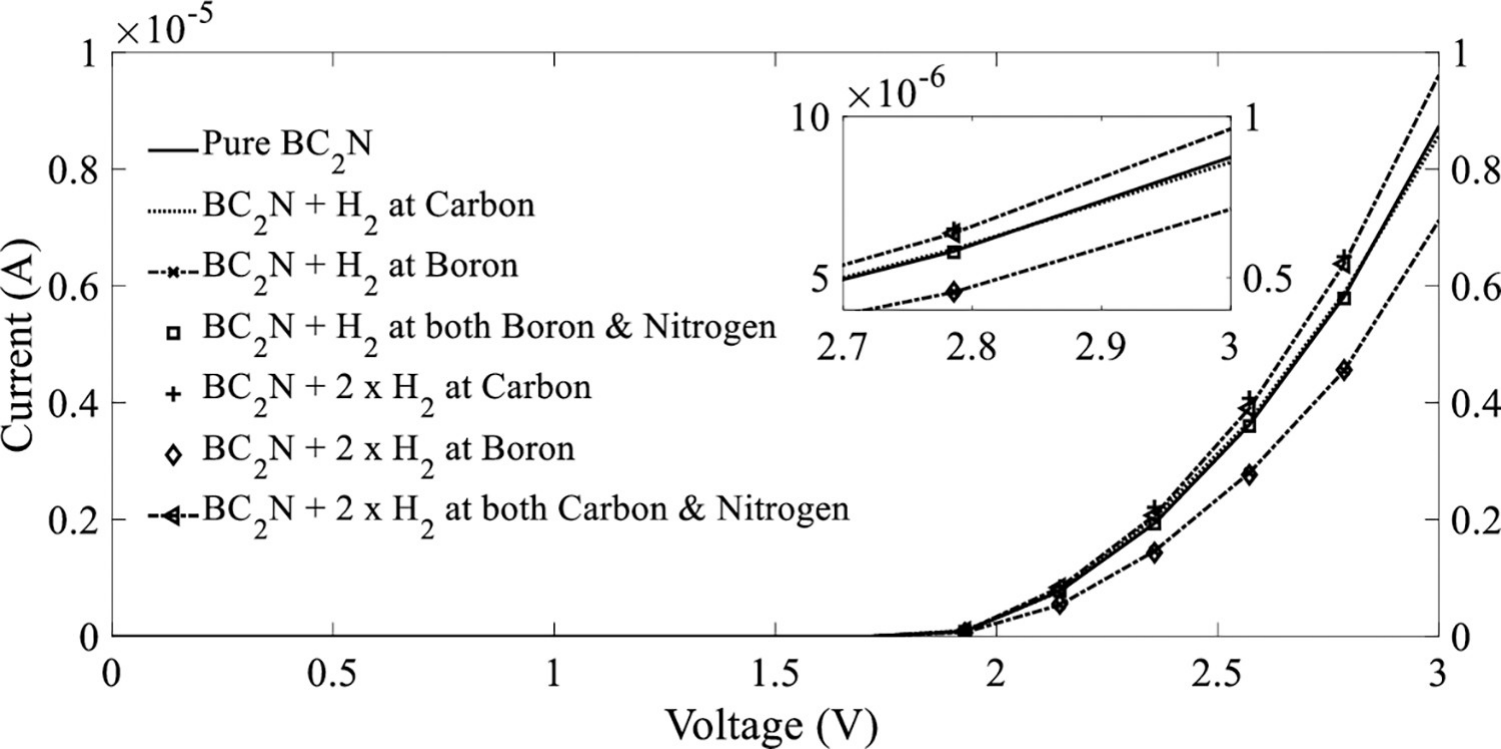
Current–voltage (I–V) characteristics before and after the adsorption of H_2_ gas molecules on BC_2_N at a temperature of 298 K.

As depicted in Fig 14, the I–V curves of BC_2_N exhibited a clear nonlinear behavior. Before the adsorption of the H_2_ gas molecules, the current passing through BC_2_N was 8.74 μ*A* for a bias voltage of 3 *V* at temperature of 298 *K*. After adsorption, the current for the bias voltage of 3 *V* at the three positions that were attached to the top of BC_2_N exhibited a considerable reduction. After the adsorption of H_2_ gas molecules, the carbon and both boron and nitrogen atoms exhibited less change in current, which were 8.58 μ*A* (1.83%) and 8.55 μ*A* (2.17%), respectively. The highest current reduction of 18.54% (7.12 μ*A*) was observed at the position of boron (Table 3). Therefore, the results verified that the detection of H_2_ gas molecules by BC_2_N was not sensitive at room temperature.

For further evaluation, a certain concentration of H_2_ molecules was added to BC_2_N under room temperature. The position of attachment was randomly selected at the top of carbon, boron, and carbon and nitrogen in BC_2_N (Figs 5–7). The corresponding I–V curves are plotted together shown in the inset of Fig 14 above. The adsorption of the added H_2_ molecules affected the performance of BC_2_N as a hydrogen sensor. The molecules positioned on carbon and both carbon and nitrogen exhibited the highest currents with 9.83 and 9.61 μ*A*, respectively, at a bias voltage of 3 *V*; the sensitivity was increased by approximately 12.47 and 9.95%, respectively. Although the H_2_ molecules were added at different locations on the boron atom in BC_2_N, the I–V curves indicated a significant reduction in current to 6.79 μ*A* (22.31%) when compared to other atoms. This implied that boron in BC_2_N was not sensitive for the detection of H_2_ molecules, and hence not preferable.

**Fig 15:**
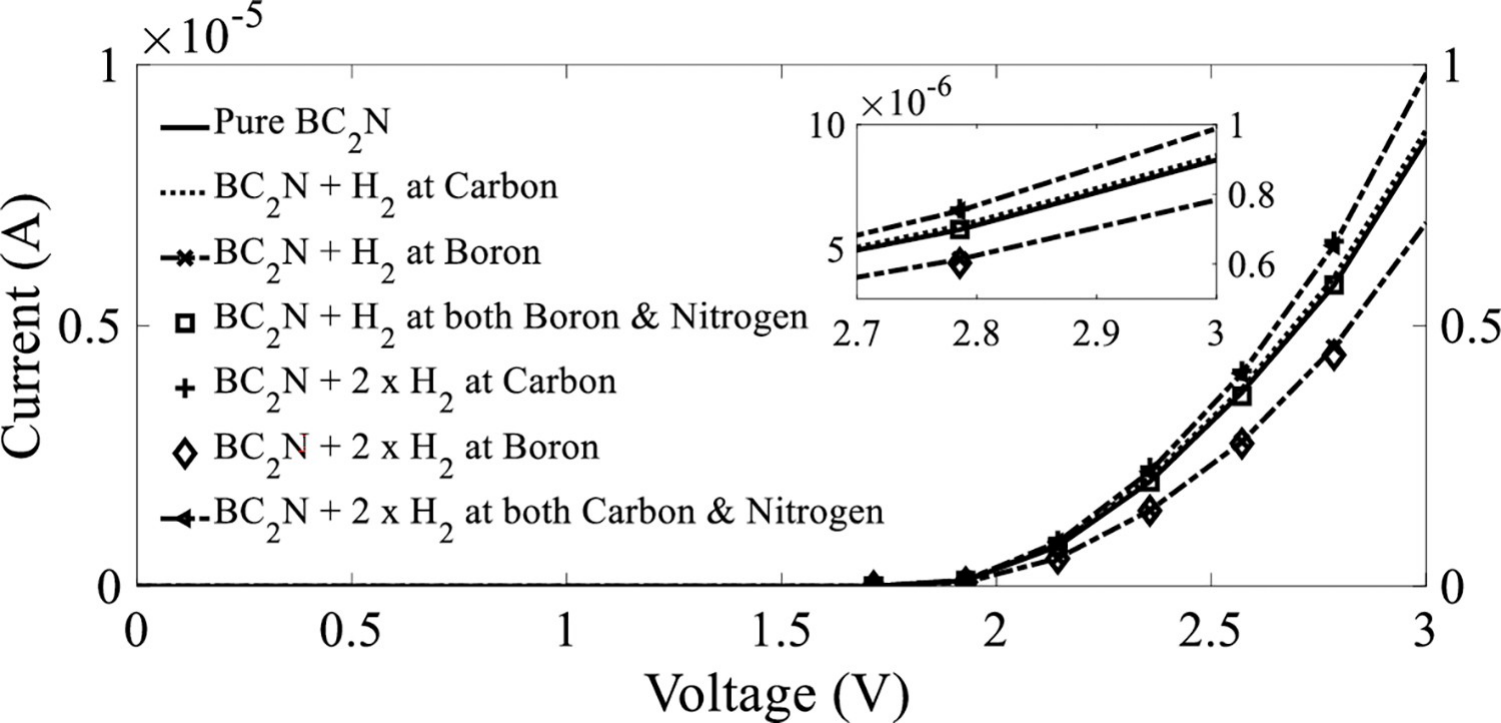
Current–voltage (I–V) characteristics before and after the adsorption of H_2_ gas molecules on BC_2_N at a temperature of 500 K.

As indicated in Fig 15, the I–V curves of BC_2_N exhibited a clear nonlinear behavior. A slight increase in current was achieved for the H_2_ molecules positioned only on carbon when the applied bias voltage was greater than 2 *V*. According to the sensitivity of the three positions of H_2_ molecules at 500 *K* (Table 3), the current passing through BC_2_N was 8.58 μ*A* at a bias voltage of 3 *V* before the adsorption of H_2_ gas molecules. However, owing to the adsorption of H_2_ gas molecules located on the carbon atom, the current was increased to 8.75 μ*A* by approximately 2% at a bias voltage of 3 *V*. A significant reduction in current was observed in the case of H_2_ gas molecules positioned on boron; herein, the current was reduced to 6.97 μ*A*, which was approximately 18.76%, at the same bias voltage of 3 *V*. Similar behaviors were observed with a current reduction of approximately 1.63% (8.44 μ*A*) after the adsorption of H_2_ gas molecules located on both boron and nitrogen atoms. Considering the I–V characteristics, the obtained results indicated that H_2_ gas molecules located on carbon exhibit high selectivity in comparison with the other two positions.

Furthermore, a certain concentration of H_2_ molecules was added to BC_2_N at a temperature of 500 *K*. As depicted in Fig 15, the current at a bias voltage of 3 *V* was increased to 9.84 μ*A* by 14.69% at both carbon and nitrogen atoms. This was followed by an increase in current for the molecule positioned on the carbon atom, with an approximate increase of 12.59% (9.66 μ*A*). Conversely, the current in the case of the molecule positioned on the boron atom exhibited a comparatively lower increment to only 6.73 μ*A* (21.56%).

**Fig 16:**
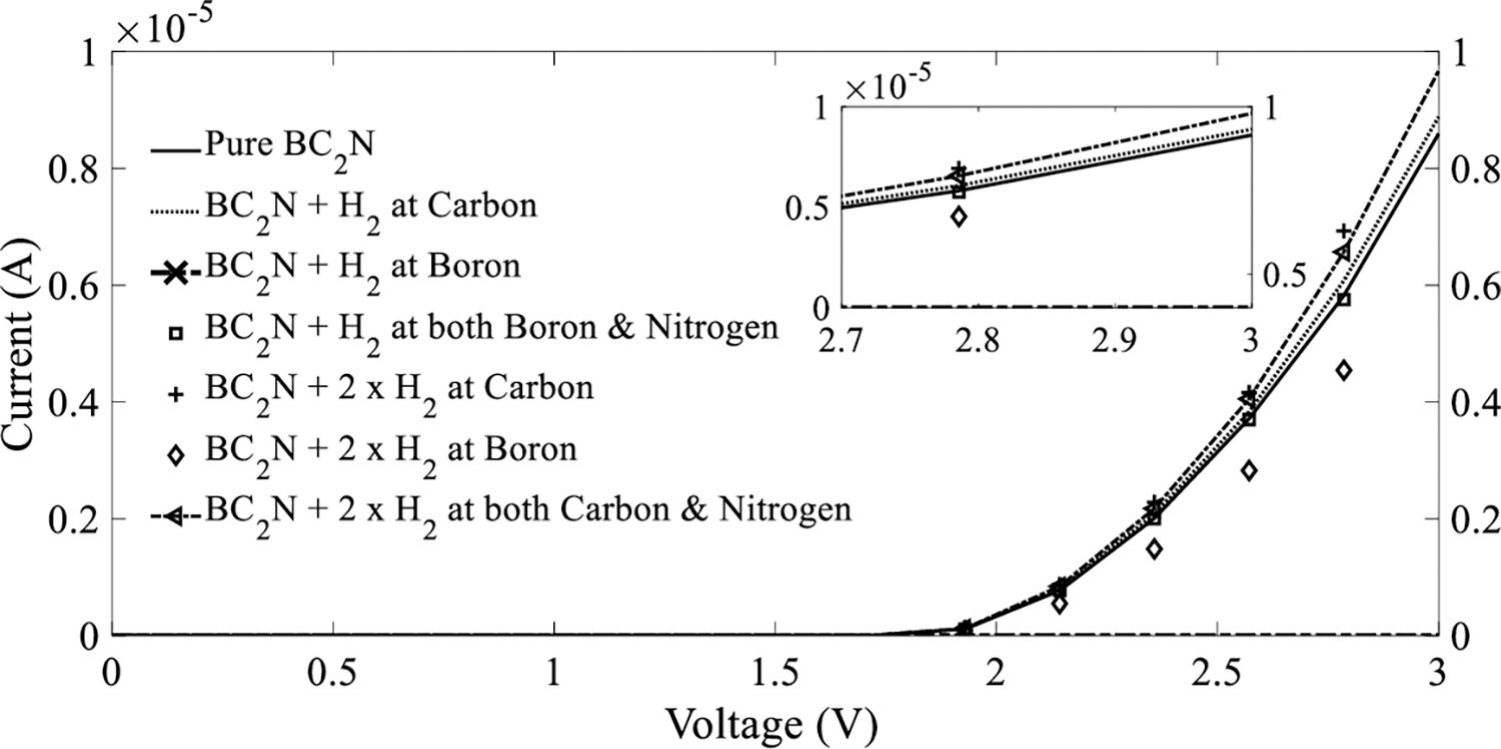
Current–voltage (I–V) characteristics before and after the adsorption of H_2_ gas molecules on BC_2_N at a temperature of 1000 K.

Finally, H_2_ gas molecules detection was tested at a temperature of 1000 *K*; Fig 16 depicts the I–V characteristics before and after the addition of molecules. The trend of I–V curves at 1000 *K* was similar to that observed at 500 *K*. The I–V curves of BC_2_N exhibited an apparent nonlinear behavior. The current passing through BC_2_N before adsorption of H_2_ gas molecules was 8.59 μ*A* at a bias voltage of 3 *V*. The I–V curves of the adsorbed H_2_ gas molecules located on the carbon atom indicated that the current was marginally increased to 8.88 μ*A* (3.38%) compared to that before adsorption. This was followed by a slight reduction in current to 8.48 μ*A* (1.3%) on both boron and nitrogen atoms. The highest reduction in current of 2.46 μ*A* (71.36%) was observed for the H_2_ molecule positioned on the boron atom. The I–V curves of H_2_ gas molecules on boron is not depicted in Fig 16 owing to the significant reduction in current.

To further maximize the potential of BC_2_N as a gas sensor, a certain concentration of H_2_ molecules was added to BC_2_N at a temperature of 1000 *K*; Fig 16 depicts the corresponding I–V curves. After H_2_ molecules were added, carbon and both carbon and nitrogen atoms recorded the highest currents of 9.88 μ*A* (15.02%) and 9.67 μ*A* (12.57%), respectively, at a bias voltage of 3 *V*. However, the added concentration of H_2_ molecules to boron resulted in a major current reduction of 6.78 μ*A* (21.07%); this indicated that the position on boron was not sensitive, because the current passing through BC_2_N before adsorption of H_2_ gas molecules was 8.59 μ*A*.

## 4. Conclusions

In this study, the sensing mechanism of hydrogen gas on BC_2_NNR was investigated using computational simulations. Three different temperatures were considered for the analysis; the same trend was observed in all cases, wherein the strongest binding site was achieved at high adsorption energies with high charge transfers. In the case of elevated temperatures of 298 and 1000 *K*, the preferable binding site was at the boron atom, whereas, for a temperature of 500 *K*, the preferable binding site was observed at the carbon atom. Based on the analysis of the I–V characteristics observed at the three temperatures, the adsorption of H_2_ gas molecules located on boron atoms was determined to be the least sensitive; particularly, the sensitivity was the lowest (71.36%) at 1000 *K*. After the adsorption of H_2_ gas molecules at room temperature, BC_2_N as a sensor was no longer sensitive. However, further addition of H_2_ gas molecules at room temperature exhibited a remarkable increment in sensitivity. Herein, the sensitivities of H_2_ gas molecules located on carbon and both carbon and nitrogen atoms increased by 12.47 and 9.95%, respectively. Furthermore, the trends of I–V curves were identical at elevated temperatures of 500 and 1000 *K* before adding more concentrations of H_2_ gas molecules. The highest sensitivity was observed in the carbon atom, whereas the boron and both boron and nitrogen atoms were not sensitive. Moreover, after adding further concentration of H_2_ gas molecules, the carbon and both carbon and nitrogen atoms exhibited dominant sensitivity at all elevated temperatures. The most insensitive detection of BC_2_N as a hydrogen sensor before and after adding concentrations of H_2_ gas molecules was identified on the boron atom.
